# Use of the Purse-String Suture to Conservatively Manage a Cornual Ectopic Pregnancy

**DOI:** 10.7759/cureus.14249

**Published:** 2021-04-01

**Authors:** Oluwatofunmi Oshodi, Jose Castaneda

**Affiliations:** 1 Obstetrics and Gynecology, Florida Atlantic University Charles E. Schmidt College of Medicine, Boca Raton, USA; 2 Obstetrics and Gynecology, Bethesda Hospital East, Boynton Beach, USA

**Keywords:** ectopic pregnancy, cornual pregnancy, salpingectomy, tubal pregnancy, vaginal spotting

## Abstract

We report the successful management of a 31-year-old female, treated by cornual wedge resection. The patient suffered from vaginal spotting and lower abdominal pain. Transvaginal ultrasonography revealed a 4-5 cm right cornual pregnancy and beta-human chorionic gonadotropin was measured to be 614.7 IU/L. This ectopic pregnancy was removed via a laparotomy with cornual wedge resection and right salpingectomy.

## Introduction

Cornual (interstitial) pregnancy is a rare form of ectopic pregnancy and accounts for only 2%-4% of all tubal pregnancies, yet has a maternal mortality of 2%-2.5% [1]. A cornual pregnancy is a pregnancy that is implanted in the proximal part of the fallopian tube, lying with the muscular wall of the uterus [2]. This section of the fallopian tube is thick and highly vascularized; thus, rupture presents later and with more severe bleeding compared to those of other ectopic pregnancies, leading to catastrophic hemorrhage [3]. The typical rupture of cornual pregnancies usually occurs later than nine weeks and can occur as late as 20 weeks [4]. The mortality rate for a cornual pregnancy is seven times greater than that of other forms of ectopic pregnancy [5]. Risk factors for cornual pregnancy include history of ectopic pregnancy, rudimentary horn, in vitro fertilization, and ipsilateral salpingectomy [1,6]. Clinical findings specific for ectopic pregnancy include the absence of an intrauterine gestational sac and beta-human chorionic gonadotropin (hCG) levels higher than 1,500 mIU per mL [7]. Transvaginal ultrasound (TVUS) has been the mainstay tool used to diagnose interstitial pregnancies (IPs), while MRI may be used in patients who are clinically stable and whose diagnosis remains unclear despite having a TVUS. Historically, IPs have been managed with the use of wedge resection by either laparoscopic or open surgery. Hysterectomies have also been used to manage IPs [8]. Current management of cornual pregnancies is less invasive, and centered on limiting hemorrhage and improving long-term fertility and obstetric outcomes. We report a case of a 31-year-old woman, who was diagnosed preoperatively with a cornual pregnancy via TVUS and a positive beta-hCG. This ectopic pregnancy was removed via a laparotomy with cornual wedge resection and right salpingectomy. The encircling suture method was used to remove the ectopic pregnancy, which has been shown to be simple, safe, effective, and nearly bloodless [9].

## Case presentation

**Figure 1:**
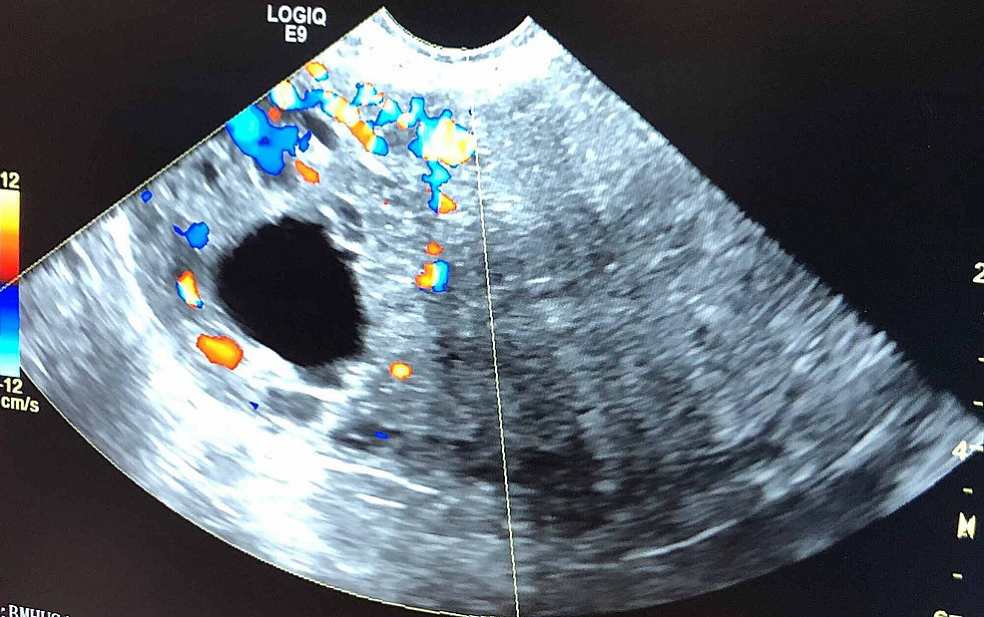
An ultrasound image of the cornual pregnancy

This is a case of a 31-year-old Hispanic female, gravida 4 para 0 aborta 3, at five weeks gestation with no significant past medical history, who presented to the emergency department (ED) after her initial prenatal visit revealed the possible presence of a cornual pregnancy on ultrasound (Figure 1). Her physician sent her to the ED for an emergent removal of the ectopic pregnancy. Her chief complaint was vaginal spotting and lower abdominal pain for the duration of one day. Clinically, the patient appeared hemodynamically stable, with a heart rate of 81 bpm and blood pressure of 130/81. Quantitative beta-hCG was 614.7 and TVUS revealed an anteverted uterus that appears grossly homogeneous. Endometrial stripe measured up to 7 mm in thickness with no gestational sac noted within the midline uterine fundus or body. In the right adnexa there was a 4.9 x 4.2 x 2.2 cm thick rind from the ovary. The location and appearance were concerning for a cornual pregnancy/IP given the positive beta-hCG and empty uterine cavity. The right ovary measured approximately 3.5 x 2.1 x 2.8 cm and contained a simple follicle. The left ovary measured 2.9 x 1.5 x 1.4 cm and appeared grossly normal. No pelvic free fluid was identified. The patient underwent emergency removal of the ectopic pregnancy.

 

Treatment and follow-up

**Figure 2:**
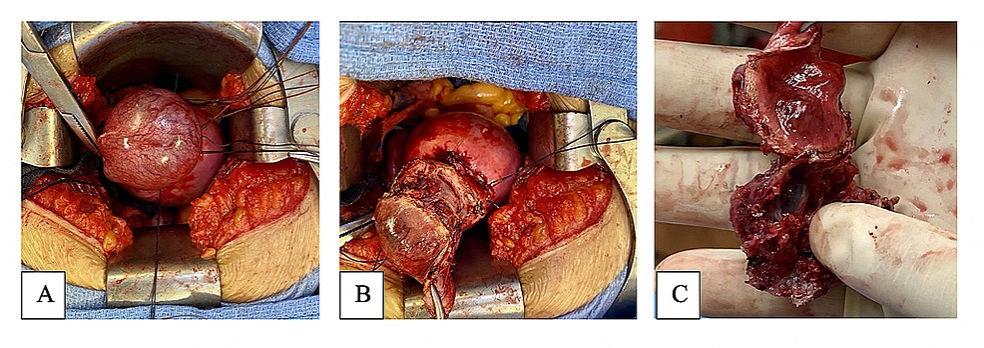
Tourniquet purse suture around right cornual mass (A), incising the mass (B), mass after excision (C)

We identified a 4-5 cm cornual ectopic pregnancy (Figure 2). The fimbria and round ligament were cut on the right side, completing a total right salpingectomy to avoid reoccurrence, and a vicryl zero suture was put in the upper part of the uterine artery three times for hemostasis. Another suture was put in medially, followed by another suture anteriorly and posteriorly in the uterine wall, around the base ectopic pregnancy. The cornual was incised and the conceptus was extracted using an encircling suture around the base of the cornual pregnancy (Figure 2A). The encircling suture was tied to produce a tourniquet effect. While tension was kept on the knot, electric cauterization was used to incise the cornua and remove the conceptus. This procedure of using encircling sutures to produce a tourniquet around the ectopic pregnancy leads to secure hemostasis. The patient’s estimated blood loss was less than 75 mL. It is routine that after surgery, hCG should be measured multiple times at different time periods to ensure the efficacy of the therapy. On post-operative day 2, her quantitative beta-hCG was measured to be 65.9 IU/L. Patient followed up with her provider for post-operative visits and quantitative beta-hCG measurements.

## Discussion

Cornual pregnancies represent 2%-4% of all ectopic pregnancies [1]. The three diagnostic criteria for cornual pregnancy described by Timor-Tritsch et al. include (1) an empty uterine cavity, (2) a chorionic sac seen separately and >1 cm from the most lateral edge of the uterine cavity, and (3) a thin myometrial layer surrounding the gestational sac [10]. The findings of our patient were consistent with all of these criteria. Rupture of a cornual pregnancy may result in intra-abdominal bleeding, hence the urgency of treatment. However, treatment of this clinical presentation still raises the concern of severe hemorrhage due to the highly vascularized region of cornual pregnancies and later time of diagnosis [3]. Management of a cornual pregnancy is dependent on the size of the ectopic pregnancy. A cornual pregnancy of medium size (<5 cm) can be managed conservatively with methotrexate if there are no contraindications, such as intra-abdominal bleeding and concomitant intrauterine pregnancy. However, treatment with methotrexate has been associated with a failure rate as high as 65% [1,8,11,12,13]. Large cornual pregnancies of 5 cm or larger should be managed surgically due to increased risk of rupture. This patient’s cornual pregnancy of 4-5 cm was concerning for rupture and thus was managed surgically through the use of a tourniquet purse-string suture. This technique not only aids in excision and minimizes blood loss, but also preserves fertility [14].

## Conclusions

Early diagnosis and treatment are of significant importance in managing cornual pregnancies as cornual pregnancies are at risk of rupture. This rupture presents later and with more severe bleeding compared to those of other ectopic pregnancies, leading to catastrophic hemorrhage. Management of cornual pregnancies is centered on limiting hemorrhage. The tourniquet purse-string suture technique aids in removal of this pregnancy, as well as minimizes blood loss and preserves the patient’s fertility.
